# The impact of enhancing vascular access care quality through monitoring and training: A multicentre observational study

**DOI:** 10.1177/11297298241296163

**Published:** 2024-11-24

**Authors:** Sonia Casanova-Vivas, Pablo García-Molina, Pablo López-Guardiola, María José Gil-Carbonell, Enrique Bernardo Hevilla-Cucarella, Vicenta Solaz-Martínez, Beatriz Valdelvira-Gimeno, Alicia Fernández-Martínez, José Vicente Visconti-Gijón, M Luisa Ballestar-Tarín

**Affiliations:** 1 Nursing Department, Faculty of Nursing and Podiatry, University of Valencia, Valencia, Spain; 2 Health Department, Valencian School of Health Studies, Valencia, Spain; 3 Associated Care Research Group, INCLIVA Foundation, Valencia, Spain; 4 Nursing Unit for Ulcers and Complex Wounds, University of Valencia Clinical Hospital, Valencia, Spain; 5 Health Department, General Hospital of Castellon, Castellon de la Plana, Spain; 6 Health Department, University Hospital of Torrevieja, Torrevieja, Alicante, Spain; 7 Health Department, Health Information Systems Analysis Service, Valencia, Spain; 8 Health Department, Arnau de Vilanova Hospital, Valencia, Spain; 9 Health Department, Virgen de los Lirios Hospital, Alcoy, Alicante, Spain; 10 Health Department, Francesc de Borja Hospital, Gandia, Valencia, Spain; 11 Nursing Department, Nursing Care and Education Research Group (GRIECE), GIUV2019-456, University of Valencia, Valencia, Spain

**Keywords:** Vascular access devices, nursing staff, hospital, quality indicators, health care, training programmes, quality improvement, data collection

## Abstract

**Aim:**

This study evaluates the impact of monitoring a primary quality indicator and the effect of training on improving the care and maintenance of vascular access devices.

**Design:**

A prospective, quasi-experimental, multicentre study with 10 periodical cross-sections from 2017 and 2020.

**Participants:**

Adult patients hospitalised over 24 h, with or without vascular access device, excluding those in emergency, psychiatry, outpatients or minor surgery units.

**Methods:**

The study included 10 cross-sections between 2017 and 2020 in all participating hospitals, using the INCATIV^®^ (Quality Indicators in Intravenous Therapy and Vascular Access) questionnaire. Training sessions for nursing staff were conducted between cross-sections, involving face-to-face sessions in open classrooms and clinical sessions in the units, based on the study Care bundle.

**Results:**

53,991 vascular accesses were analysed, with an average INCATIV Index score of 8.95 (SD: 1.32), showing improvement from 8.09 in the first cross-section to 9.21 in the last. Significant variability was observed between hospitals, with secondary hospitals scoring lower on the INCATIV^®^ Index. Training interventions 1 and 2 showed significant improvement across all hospital categories, whereas intervention 5 did not show significant effects. Compliance with main recommendations was studied, with a notable decrease in phlebitis rates from 4.45% in the first cross-section to 1.23% in the tenth.

**Conclusion:**

The study developed a single indicator to assess and quantify vascular access care quality. It demonstrated that implementing a care bundle through serial training interventions and continuous assessment by nursing staff, supported by process indicators and data availability on the study platform, significantly reduces complications and enhances the quality and safety of vascular access care.

## Introduction

Globally, vascular access (VA) management is an essential part of modern healthcare^1^; suitable training and assessment are critical to ensuring quality care. This is why healthcare institutions must standardise processes and procedures for the selection, insertion and care of vascular access devices in patients requiring them through comprehensive management programmes. These programmes should ensure optimal, highly durable vascular access with minimal adverse events that affect patient safety.^1^

The recommendation for indicator monitoring is included in most of the latest global clinical practice guidelines on the prevention of catheter-related infections.^2–6^ Also, quality standards for vascular access care are included but there is still limited capacity to evaluate or compare how safe vascular access care is.^7^

Nursing care should be aimed at reducing complications and making catheter insertion a minimal-risk technique, assuming multicomponent care packages, or care bundles and evidence-based best practices. More attention needs to be paid to the quality of the process, as this control will directly affect the quality of the results.^8^

Several studies over the last decade^4,9,10^ have demonstrated the efficacy of these bundles in improving quality; nonetheless, few of these studies have been able to quantify and compare the compliance with these recommendations and their evolution over time.

In Spain’s Valencian Community, the INCATIV^®^ Health Working Group, a nursing research team, initiated the INCATIV^®^ Quality Indicators in Intravenous Therapy and Vascular Access Study in 2008. This study led to the creation of a management tool aimed at enhancing the quality of care for adult patients.

Between 2008 and 2013, the tool allowed the periodical monitoring of a series of indicators using a data collection questionnaire (INCATIV^®^ Questionnaire) on the characteristics of the state of vascular accesses in hospitalised patients and the calculation of a standard reference variable to assess whether vascular access care was optimal or non-optimal. In addition, during the monitoring process, training interventions aimed at nursing professionals were given based on the INCATIV^®^ care bundle.^11^ At the end of this study in 2016, the research management group (MG) set out to identify and weigh the variables that could most influence good vascular access care via an explanatory model.^12^

This study aimed to assess the impact of monitoring indicators through a main quality indicator and the impact of training on improving the quality of care and maintenance of vascular access devices (VAD).

## Methods

A quasi-experimental study with 10 cross-sections in each of the participating hospitals between 2017 and 2020. The study was designed by the MG, comprising 11 nursing professionals with different management, research and care profiles. An online web platform (www.incativ.org) was designed for data management and registration. In each hospital, a researcher’s team was created headed by a coordinator and field researchers, who participated in the collection and data uploading.

### Inclusion/exclusion criteria

Vascular access requirements of all patients admitted through the 33 participating hospitals were evaluated. These were classified according to hospital level as per resources and healthcare capacity^11^ with 4 primary hospitals, 13 secondary hospitals, 11 tertiary hospitals and 5 specialised hospitals participating. The inclusion criteria were adult patients admitted for more than 24 h, excluding those admitted to emergency units, psychiatry, outpatients or minor surgery.

### Data collection

Data collection was carried out by the field investigators, who were approved for data collection through prior training given by the coordinating researchers of each hospital avoiding any observer bias. The INCATIV^®^ Questionnaire, used in the previous study,^12^ was completed and uploaded the data onto the application, which automatically calculated the indicators based on all the variables collected.

**Figure 1.**
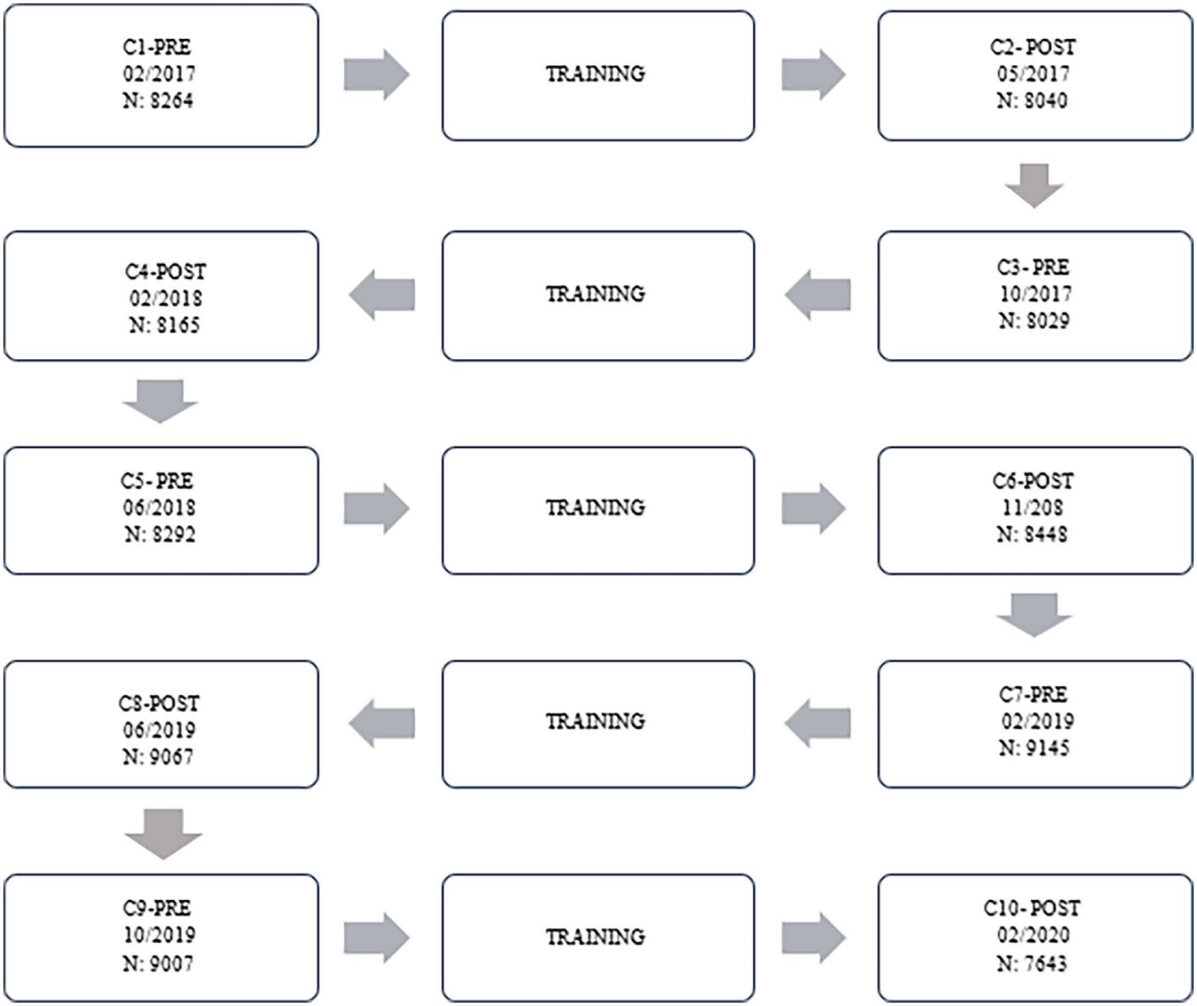
Cross-sections and training interventions flowchart.

Ten cross-sections were performed according to the timeline (Figure 1). Five sections were pre-training interventions and another five were post-training interventions.

### Instrument and variables

The aforementioned INCATIV^®^ Questionnaire consisted of 22 items. The indicators calculated on the platform were occupancy rate, intravenous therapy use rate and vascular access device-related rates that is, type of access routes, type of prescription, use of the device, type of infusion, type of system, anatomical location, type of dressing, dressing date record, post-catheter access, state of catheters, origin of venipuncture by services and phlebitis rate. The indicators were shown in bar graphs and could be consulted by hospitals or by the services of each hospital.

A panel of experts was created, and utilising Delphi methodology, designed a multi-component indicator tool, called the INCATIV^®^ index. The index was constituted with the variables resulting from the explanatory model^13^ developed by the MG group in a previous stage. To facilitate interpretation, the panel of experts agreed on the assignment of weights to each variable to obtain a value between 0 and 10. This score was used to assess each of the vascular accesses and, thus, obtain the global mean for each hospital and each service. The variables comprising the index are shown in Table 1.

**Table 1.** INCATIV^®^ Index variables. Operational definitions.

Variables	Operational definition
Appropriate dressing	The dressing used was transparent, or in the case of patient perspiration, gauze was used.
Date recorded on the dressing	The date the catheter was inserted was labelled and visible on the dressing.
Closed system	There was no open access either at the connection to the catheter or throughout the infusion system.
Visible insertion point	The catheter insertion site was perfectly visible.
Last dressing change within last 7 days	The dressing had been changed within the last 7 days.
Adequate dressing conditions	The dressing was well adhered to the skin, not peeled off, dry, not wet and the dressing was seen to be kept clean.
Presence of phlebitis	Presence of induration, pain and/or signs of inflammation at the insertion site or catheter.

### Study interventions

The training interventions were designed according to the latest scientific evidence by the study MG and transferred to each coordinator. The training interventions were given to nursing care professionals related to the care and maintenance of vascular accesses devices. The interventions were 1-h sessions in an open classroom (available during the morning shift in different sessions to facilitate the attendance of all nurses during their shift), or on-site clinical sessions in the hospital units, 1 month before each post-intervention cut-off (C2, C4, C6, C8 and C10).

The training content was based on the INCATIV^®^ care bundle, with the emphasis on the index’s variables and the analysis of the results of the previous pre-intervention cross-section of each hospital in order to detect points for improvement necessary to reinforce the recommendations, as well as on the algorithms for choosing adequate VAD, maintenance and resolution of complications.

### Data analysis

To analyse the results of the study, platform database was used with the records of the 10 cross-sections carried out between 2017 and 2020. A global descriptive analysis was performed based on frequency and percentage tables. The INCATIV^®^ Index was calculated for each access and according to each hospital, pre- and post-intervention. To analyse the relationship between categorical variables, the chi-squared test was used, and for quantitative variables (INCATIV^®^ index), normality was ruled out by the Kolmogorov-Smirnov and Kruskal-Wallis *H* tests. INCATIV^®^ Index variables evolution between cross-sections was performed with ANOVA. The statistical programme used was the SPSS 28.0 software package, licensed by the University of Valencia, and Microsoft^®^ Excel^®^ for Microsoft 365 MSO, v. 2408. A *p*-value <0.05 was considered significant for all tests.

### Ethical considerations

This project adhered to the recommendations established in the Declaration of Helsinki and subsequent amendments, complying with data protection laws. An exemption from the request for informed consent was requested and approved by the Research Ethics Committee of the Department of Health of La Ribera CCE04072018. Each centre’s results report could be downloaded freely but each hospital was identified with a numerical code, allowing its anonymity.

## Results

In the 10 periods studied, a total of 84,100 registers were collected from the total number of hospital beds observed, categorised as occupied bed, empty bed and occupied bed with intravenous therapy.

A total of 420 nursing professionals collected these data from the 33 participating hospitals. The occupancy percentage of the participating hospitals was 76.46% and the rate of patients with VA in occupied beds was 83.96%.

The number of VAD observed during this study period was 53,991. In each cross-section, an average of 5000 VAD was registered. Of these, 54.42% were evaluated in tertiary hospitals, 37.42% in secondary hospitals, 4.87% in primary hospitals and 3.27% in specialised hospitals. The distribution of the registers by cross-section is shown in Table 2.

**Table 2.** Number of registers by type of hospital and cross-section.

Cross-sections (C)										
Hospital type	C1-Pre	C2-Post	C3-Pre	C4-Post	C5-Pre	C6-Post	C7-Pre	C8-Post	C9-Pre	C10-Post
Primary	315	244	253	326	267	273	287	272	267	123
Secondary	2255	1990	1989	2186	1886	2000	2150	1991	2016	1755
Tertiary	2871	2593	2483	2879	2716	2835	3569	3315	3360	2762
Specialised	199	178	208	195	155	167	195	144	146	176

Table 3 shows the sociodemographic data and variables of the questionnaire. The vascular accesses evaluated belonged to men in 55.4% of cases and 44.6% to women. The mean age of the patients was 67.27 years (SD = 16.82). The short peripheral catheter (SPC) was the most frequent vascular access (82.1% of cases), with vascular access devices being mostly for intermittent use and 40% for drug administration.

**Table 3.** Description of variables.

Variable	Category	*N*	%
Cross-sections	1	5640	10.4
	2	5005	9.3
	3	4933	9.1
	4	5586	10.3
	5	5024	9.3
	6	5275	9.8
	7	6201	11.5
	8	5722	10.6
	9	5789	10.7
	10	4816	8.9
		53,991	100
Sex	Men	29,935	55.4
	Women	24,056	44.6
Age	67.27 (±16.82)		
Catheter type	Short peripheral catheter	44,683	82.80
	Subcutaneous catheter	704	1.30
	Central venous catheter	3828	7.10
	PICC	2406	4.50
	Midline	177	0.30
	Arterial catheter	1376	2.50
	Chest port	808	1.50
	Long peripheral catheter	9	0.00
Vascular access prescription	Parenteral nutrition	1566	2.9
	Medication	21,872	40.5
	Fluid therapy	17,969	33.3
	Blood products	137	0.3
	Monitoring	813	1.5
	Unidentified/unlabelled	262	0.5
	None (in disuse)	11,372	21.1
Use of the line	Continuous	22,778	42.2
	Intermittent	29,368	54.4
	In Y	1845	3.4
Type of infusion	Gravity infusion	25,434	47.1
	Pump	9633	17.8
	Elastomeric infusion system	1275	2.4
	None	17,649	32.7
System type	Closed	43,562	80.7
	Open	10,429	19.3
Anatomical location	Back of the hand	13,227	24.5
	Wrist	7721	14.3
	Forearm	16,029	29.7
	Arm flexure	9179	17
	Upper third of the arm	2318	4.3
	Subclavian	1309	2.4
	Jugular	1869	3.5
	Chest	691	1.3
	Femoral	498	0.9
	Head	2	0
	Foot	146	0.3
	Tunnelled	273	0.5
	Other	729	1.4
Type of dressing	Opaque dressing	3752	6.9
	Clump	1757	3.3
	Transparent dressing	37,021	68.6
	Clear dressing with chlorhexidine GCH	1086	2
	Dressing with GCH-impregnated disc	278	0.5
	Clear dressing with reinforced border	9929	18.4
	Port (intermittent)	168	0.3
Adequate dressing conditions	Yes	45,009	83.4
	No	8728	16.2
	Port (intermittent)	254	0.5
Change of dressing within the last 7 days	Yes	53,068	98.3
	No	923	1.7
Visible insertion point	Yes	45,414	84.1
	No	8231	15.2
	Port (intermittent)	346	0.6
Insertion date on the dressing	Yes	32,383	60
	No	21,504	39.8
	Port (intermittent)	104	0.2
Post-catheter access	Needle free connector (NFC)/safety stopcock	7884	14.6
	Perforating membrane stopcock	320	0.6
	3-way stopcock WITHOUT extension	2224	4.1
	3-way stopcock WITHOUT extension WITH NFC	1421	2.6
	Extension WITH 3-way stopcock	9032	16.7
	Extension WITH 3-way stopcock WITH NFC	15,039	27.9
	Mono/Bi/tri lumen extension	1623	3
	Bifurcated extension Mono/bi/trifurcated WITH NFC	15,876	29.4
	Port (intermittent)	187	0.3
	Other	385	0.7
Three-way stopcocks	One stopcock	25,522	47.3
	No stopcock	24,337	45.1
	More than one stopcock	3752	6.9
	Port (intermittent)	380	0.7
Phlebitis	Yes	970	1.8
	No	53,021	98.2

**Figure 2.**
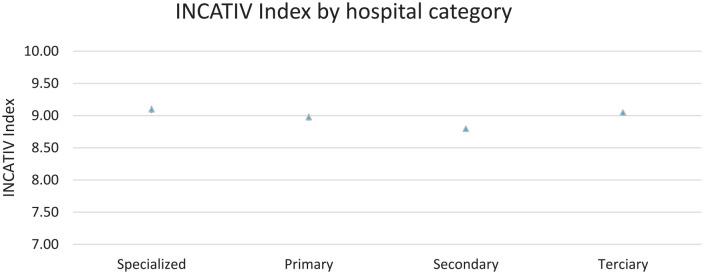
INCATIV^®^ Index by type of hospital.

The overall results of the INCATIV^®^ Index for all vascular accesses studied had a mean score of 8.95 (SD: 1.32) out of a maximum of 10. Differences were observed when analysing by hospital category, with hospitals classified as secondary showing lower INCATIV^®^ Index scores (Figure 2). There were no significant differences according to sex.

### Effects of training interventions

During the period analysed, 50 field researchers were responsible for providing the training in its different modalities. Between 80% and 90% of nurses from all hospitals in each training intervention were trained during the study.

**Figure 3.**
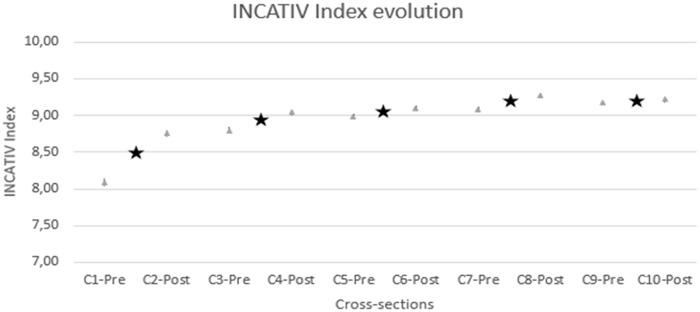
Evolution of the INCATIV^®^ Index. Black stars show interventions between sections.

The INCATIV^®^ Index evolved favourably from the first cross-section to the last, from 8.09 to 9.21 (Figure 3). The bivariate section-by-section analysis showed how the INCATIV index evolved incrementally and significantly between the sections in which there was a training intervention (i.e. C1→C2; C3→C4; C5→C6 and C7→C8). In contrast, between the sections with no training (C2→C3; C4→C5; and C6→C7), and between C9 and C10, there were no significant variations, with a 95% confidence interval.

When analysing in detail, the INCATIV^®^ index variation from C1-Pre to C2-Post was 0.613 points. This improvement is attributed to the first intervention and this difference is significant (*p* < 0.001).

Table 4 summarises the effect of these interventions by hospital category, Effect of training interventions 1 and 2 were significant on all categories (*p* *<* 0.001). Conversely, intervention 3 is only significant in secondary hospitals and intervention 4 in secondary and tertiary hospitals. The effect of training intervention 5 was not significant in any hospital group (*p* > 0.04).

**Table 4.** Effect of the trainings interventions (TI) on INCATIV^®^ Index mean (C1-C2; C3-C4; C5-C6; C7-C8; C9-10).

Training intervention (TI)	Cross-section (C)	Specialised	Primary	Secondary	Tertiary	Total
TI1	C1-Pre	8.291	8.128	7.744	8.348	8.092
	C2-Post	8.904	9.038	8.622	8.824	8.757
		***p*** ***<*** 0.001*	***p*** ***<*** 0.001*	***p*** ***<*** 0.001*	***p*** ***<*** 0.001*	***p*** ***<*** 0.001*
TI2	C3-Pre	9.043	8.622	8.610	8.940	8.795
	C4-Post	9.143	9.098	8.915	9.114	9.036
		***p*** ***<*** 0.001*	***p*** ***<*** 0.001*	***p*** ***<*** 0.001*	***p*** ***<*** 0.001*	***p*** ***<*** 0.001*
TI3	C5-Pre	9.169	8.820	8.874	9.063	8.982
	C6-Post	9.350	9.098	9.039	9.131	9.101
				***p*** ***<*** 0.001*		***p*** ***<*** 0.001*
TI4	C7-Pre	9.230	9.220	8.946	9.138	9.078
	C8-Post	9.454	9.332	9.192	9.297	9.266
				***p*** ***<*** 0.001*	***p*** ***<*** 0.001*	***p*** ***<*** 0.001*
TI5	C9-Pre	9.433	9.4	9.045	9.226	9.169
	C10-Post	9.190	9.541	9.103	9.270	9.213

* Statistical significance with the previous cross-section.

Table 5 shows the evolution of INCATIV^®^ Index mean by bundle recommendations after training interventions. Interventions 1 and 2 are significantly effective in improving compliance with the five main recommendations.

**Table 5.** Evolution of INCATIV^®^ Index mean by bundle recommendations after the training interventions (TI).

Bundle recommendations	TI1		TI2		TI3		TI4		TI5	
	C1-Pre	C2-Post	C3-Pre	C4-Post	C5-Pre	C6-Post	C7-Pre	C8-Post	C9-Pre	C10-Post
Use of transparent dressing*	75.79	85.70	87.00	90.35	91.00	91.80	92.05	93.40	93.07	91.98
		***p*** ***<*** **0.001**		***p*** ***<*** **0.001**			***p*** ***<*** **0.001**		***p*** ***<*** **0.001**	
Record of insertion date	69.35	80.92	81.33	86.19	85.04	86.50	87.10	90.55	89.29	89.88
		***p*** ***<*** **0.001**		***p*** ***<*** **0.001**		***p*** ***<*** **0.001**		***p*** ***<*** **0.001**		***p*** ***<*** **0.001**
Visible insertion point	35.59	53.81	53.41	62.25	60.35	65.72	61.69	70.53	65.62	72.13
		***p*** ***<*** **0.001**		***p*** ***<*** **0.001**		***p*** ***<*** **0.001**		***p*** ***<*** **0.001**		
Adequate dressing conditions**	74.11	81.68	82.58	84.37	84.92	85.34	85.27	86.73	85.31	87.41
		***p*** ***<*** **0.001**		***p*** ***<*** **0.001**			***p*** ***<*** **0.001**		***p*** ***<*** **0.001**	
Type of IV system	58.60	69.65	77.38	81.95	81.79	83.51	87.34	86.28	88.34	91.26
		***p*** ***<*** **0.001**		***p*** ***<*** **0.001**		***p*** ***<*** **0.001**			***p*** ***<*** **0.001**	

* Adequate transparent dressing includes transparent dressing with CHG and reinforced borders.

** A dressing that is not wet, dirty, or displaced and appears to be in good hygienic condition.

**Figure 4.**
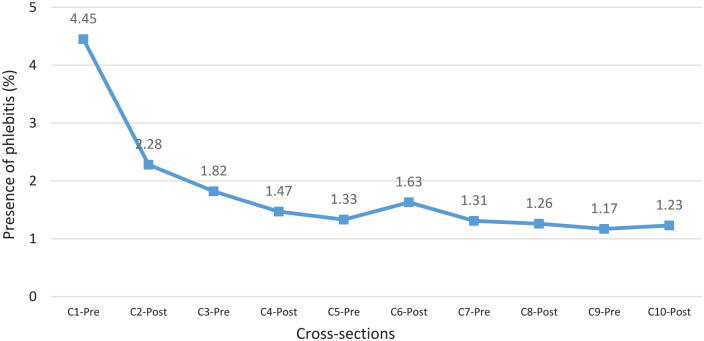
Evolution of the presence of phlebitis.

On the other hand, the incidence of phlebitis decreased significantly from 4.45% at the beginning of the study (C1-Pre) to 1.23% at the end (C10-Post; Figure 4).

## Discussion

The development of the INCATIV® Index has enabled an objective evaluation of the quality and standard of vascular access care in the adult population.

The strength of this study is that data was collected from the bedside, without consulting medical records, which allowed a high volume of data collection. The indicator allowed researchers to quantify the degree of compliance with the recommendations on vascular access care and maintenance by nursing staff and measured the impact of the training interventions carried out. The INCATIV^®^ Index was calculated on 53,991 VA evaluated, observing an overall improvement from 8.09 to 9.21 points from the initial to the last cross-section. Secondary hospitals significantly improved their INCATIV^®^ index throughout the study.

In this study, the monitoring also allowed researchers to observe that the overall evolution of quality was positive; however, there was significant variability in the application of the recommendations across hospital centres. Therefore, like other studies on strategies implemented for the care of SPCs, the need to work with protocols to homogenise care and avoid complications is highlighted.^9,14^ The heterogeneity of care causes worrying degrees of disparity in terms of quality of care.^15^ Continuous monitoring of indicators allows constant surveillance for early intervention if a reduction in the level of care quality is detected.

Another important aspect of this study is that it demonstrates how training interventions significantly influence the improvement of the variables studied. The training interventions carried out were based on the INCATIV^®^ care bundle and analysis of the previous data from each cross-section. Zingg et al.^16^ showed that a care bundle can reduce complications by standardising daily practice and, through the analysis of its own results (real-time feedback) and comparison with other hospitals, it can increase motivation.

In this study, differences were found between the two types of training that were given; face-to-face training workshops, accompanied by an analysis of previous data and focused on the weakest points susceptible to improvement, were more influential than the on-site clinical sessions. These clinical sessions were the last training interventions carried out to facilitate attendance, reduce mobility and bring the training and results closer to the professional; however, they did not obtain a significant quality increase.

Nevertheless, this reinforces the importance of continuous and specific training aimed at professionals. Recent good practice guidelines indicate that education is highly valued by health professionals and can improve their confidence and attitude.^4^ In addition, other studies coincide in applying mainly educational elements, team building, assessment and feedback.^15,17^ Furthermore, as stated in the latest guidelines from the Infusion Nurses Society,^6^ the implementation of evaluation methods to identify the clinical skills of health professionals is considered a method that can produce greater clinician satisfaction, improve confidence and increase independence.

Another strength of this study is the generated database. The availability of aggregated data provides very valuable information and allows nursing managers to make informed decisions about which units and shifts to target in upcoming training, and on which points to reinforce training.^18^

Therefore, this study has permitted to analyse adherence to the main recommendations measured by the INCATIV^®^ Index and to observe that compliance with the recommendations contained in the bundle reduces the frequency of complications.

Catheter-related complications are common and associated with clinical and economic burdens, increased hospitalisation costs, longer stays and increased risk of death among these patients.^7^ The complication evaluated in this study was phlebitis. Throughout the study, a decrease in the prevalence of phlebitis, measured through the Visual Infusion Phlebitis Score,^19^ was detected during the observations made over the 10 periods described. There was a significant reduction of 3.22% in the diagnosis of phlebitis, which may be in line with other studies that measure this complication.^20^ On the other hand, we find authors such as Arias-Fernández et al.^21^ who suggest higher rates of phlebitis (5.6%) in short SPCs, or Yasuda et al.^22^ who reported a 7.5% rate of phlebitis related to this type of catheter.

In 2023, a systematic review carried out by Xu et al.^14^ established that complying with the recommendations of a care bundle also allows optimisation of the use of resources. In this study, a slight change in trend was observed in the choice of vascular access device, even though the most prevalent vascular access observed was the SPC (82.80%). Another type of study would be necessary to verify that the application of the algorithm has been correct, following the algorithms of choice recommended in the Gavecelt and INS guidelines^6,23^ taught in the training interventions.

Choosing suitable materials to use during vascular access care and maintenance helps to minimise care-related deficiencies and immediate and late complications, for example, the choice of a transparent dressing with reinforced borders. Atay and Yilmaz Kurt,^24^ in their study, demonstrated that those patients in whom a sterile semipermeable dressing was used had a later onset of complications than those whose dressing was not sterile. In the 10 periods observed in this study, we detected a change in the use trend of these dressings, with a frequency of use increasing from 75.79% to 91.98% (*p* < .001). At the same time, the recommendation to record the insertion date on the dressing improved from 69.35% to 89.88% (*p* < .001). Several guidelines agree that not recording the date of insertion on the dressing is a bad practice.^5,25^

Also, regarding the choice of materials used post-catheter, an increase in the use of needle-free connectors instead of three-way stopcocks was observed. These maintain a closed system, avoiding manipulations and disconnections that could also cause contamination of the vascular access. In their study, Rickard et al.^26^ in 2023, stated that integrated SPCs can significantly reduce the burden of PIVC failure on patients in the health system. In this study, the use of a closed system rose from 58.60% to 91.26%, a statistically significant difference.

Another of the recommendations analysed was the visibility of the insertion point. Zingg et al.^16^ concluded that, without a daily inspection of the insertion point, it was not possible to rapidly identify the complications of a vascular access device, such as phlebitis, infiltration, mechanical failure or displacement. The latest European recommendations^23^ advise an adequate monitoring of the functioning of the device and a visual inspection of the insertion site during each nursing shift. Nurses should become more aware of daily monitoring of vascular access insertion sites, their proper functioning, the recording of the insertion date for the next shifts and maintaining vascular accesses in optimal conditions (dry, well adhered)^27^ of hygiene, cleanliness and comfort for the patient.^28^ In this study, the exit site visibility improved from 35.59% to 72.13%, a statistically significant improvement.

Milutinović et al.^27^ also stated that a poor condition of the dressing can cause colonisation by microorganisms that can lead to serious complications, therefore, it is important to maintain suitable hygiene and cleanliness conditions, as well as adequate changes, as recommended by the guidelines. In this study, compliance with this variable was high in the initial cross-section (74.11%) yet still improved significantly to 87.41% (*p* < 0.001).

## Limitations

This study has some limitations. First, there might be observer bias since, as in all facilities, it was the nursing professionals themselves who evaluated the results. However, this risk was mitigated by having the field researchers switch their work units to collect data, and the data collection dates of all sections were kept confidential. Secondly, the interventions were evaluated through prevalence assessments as it was not possible to study patients longitudinally. This was due to the complexity of a multicentre study and the increased amount of time and resources that would be needed. An incidence study would provide better indicators of the frequency of adverse effects^9^; however, prevalence studies are a true snapshot of the moment. Similar studies^29,30^ reinforce that bedside prevalence studies, before and after interventions, are an appropriate instrument to measure improvements in care compliance.

Other variables related to catheter insertion or removal or other factors that could influence the appearance of complications—adequate hand hygiene, type of drug to be administered or the patient’s own condition—were not considered; because, in this study, only variables related to the care and maintenance of vascular access devices, gathered by observation, were included.

## Conclusion

The INCATIV^®^ Index is a unique multi-variable indicator that quantifies the state of vascular access device care, enabling both intra- and inter-hospital comparisons. It helps monitor nursing compliance with key vascular access care recommendations and measures optimisation and material use changes. By implementing the bundle through serial training, continuous nursing assessments and real-time data availability on the study’s platform, complications could be significantly reduced, enhancing the quality and safety culture in vascular access care.

By this reason, this study has been also implemented in paediatric population during the years 2021 to 2023 thanks to a research project. Results are being analysed by the MG.

Further studies might include hospitals from different Spanish areas to be able to compare best practice on vascular access care and allow the design and analysis of training sessions and their content in order to plan comprehensive training national strategies and ensure that the quality of care is monitored and constantly improving.

## Supplemental Material

sj-pdf-1-jva-10.1177_11297298241296163 – Supplemental material for The impact of enhancing vascular access care quality through monitoring and training: A multicentre observational study Supplemental material, sj-pdf-1-jva-10.1177_11297298241296163 for The impact of enhancing vascular access care quality through monitoring and training: A multicentre observational study by Sonia Casanova-Vivas, Pablo García-Molina, Pablo López-Guardiola, María José Gil-Carbonell, Enrique Bernardo Hevilla-Cucarella, Vicenta Solaz-Martínez, Beatriz Valdelvira-Gimeno, Alicia Fernández-Martínez, José Vicente Visconti-Gijón and M Luisa Ballestar-Tarín in The Journal of Vascular Access
