# Supplementary material for: The impact of enhancing vascular access care quality through monitoring and training: A multicentre observational study
Source: J Vasc Access. 2024 Nov 24;26(5):1661–72. doi: 10.1177/11297298241296163 (PMC12397542; doi:10.1177/11297298241296163)
Supplement: sj-pdf-1-jva-10.1177_11297298241296163 – Supplemental material for The impact of enhancing vascular access care quality through monitoring and training: A multicentre observational study [file sj-pdf-1-jva-10.1177_11297298241296163.pdf]

Contributors list (2017-2019)

Hospital	Surname	Name	Profile
CONSORCIO HOSP PROVINCIAL CASTELLÓN (CV)	CHIVA PERIS	JOSEP VICENT	Coordinator Researcher Team
	ANDUJAR ANGULO	VERÓNICA	Field Researcher
	TALAMANTES MELIÁ	LIDON	Field Researcher
	ALBIOL MAS	CLARA	Field Researcher
	SAURA ALLEPUZ	CARMEN	Field Researcher
HOSP CLINICO UNIVERSITARIO VALENCIA (CV)	ZAFRA PIRES	Mª JESUS	Coordinator Researcher Team
	LANZON SERRA	TERESA	Field Researcher
	INAT CARBONELL	JAVIER	Field Researcher
	AGUILAR SANTAISABEL	PILAR	Field Researcher
	ALBA LUQUE	ROBERTO	Field Researcher
	AÑOVER MARTINEZ	Mª JOSÉ	Field Researcher
	GIMENO MORANT	SILVIA	Field Researcher
	MARTINEZ FUERTES	MAGDALENA	Field Researcher
	RAMON ORDOÑEZ	LUCIA	Field Researcher
	GARCIA GRANCHA	EDURNE	Field Researcher
	GINEL RAMIREZ	RAQUEL	Field Researcher
	GARCIA FERRER	LORENA	Field Researcher
	GARCIA FERRANDIS	SARA	Field Researcher
	CLEMENT PEREZ	ANA ISABEL	Field Researcher
HOSP GENERAL UNIVERSITARIO ALICANTE (CV)	ADSUAR MAS	Mª ASUNCION	Coordinator Researcher Team
	SEGURA CUENCA	MERCEDES	Field Researcher
	DOMINGO POZO	MANUELA	Field Researcher
	CLEMENT SANTAMARIA	ANA ROSA	Field Researcher
	FUSTER PEREZ	MARINA	Field Researcher
	ALEMAÑ SANTOS	VIRGINIA	Field Researcher
	IÑESTA SALCEDO	SANDRA	Field Researcher
	CREMEDES PASTOR	BEGOÑA	Field Researcher
	ORTIZ DE SARACHO PEREZ MASGORET	Mª TERESA	Field Researcher
	PAYA PEREZ	BELEN	Field Researcher
	AMOEDO ALBERO	MCARMEN	Field Researcher
	OSUNA GARCIA	CARMENOS	Field Researcher
	BIELSA JULIA	EMILIA	Field Researcher
	JIMENEZ CANTO	EMILIA	Field Researcher
	BEVIA PUCHE	GRISEL	Field Researcher
	RODRIGUEZ LOZOYA	JUANA	Field Researcher
	ALCARAZ DEL BUSTO	Mª JOSE	Field Researcher
	BAÑO GUEDEA	Mª LUISA	Field Researcher
	MOLLA SANTONJA	Mª VICTORIA	Field Researcher
	IZQUIERDO GARCIA	NIEVES	Field Researcher
	LLORENS CASTELLO	MONSERRAT	Field Researcher
	MATEO ROBLES	ANA ROSA	Field Researcher
	DE CASTRO IBAÑEZ	ISABEL	Field Researcher
	SERRANO BORRELL	ROSA	Field Researcher
	PLA SANCHEZ	ROSARIO	Field Researcher
	NAVARRO PARDO	PILAR	Field Researcher
	SALAZAR FERNANDEZ	SUSANA	Field Researcher
	NAVARRO PARDO	PILAR	Field Researcher
	QUIÑONES BUSTOS	CARMEN	Field Researcher
	SERRANO BORREL	ROSA	Field Researcher
	FLOR TOMAS	ANDREA	Field Researcher
	ABAD LLEDO	ESTEFANIA	Field Researcher
	BALBOA ESTEVE	SONIA	Field Researcher
	DECASTRO IBAÑEZ	ISABEL	Field Researcher
HOSP GENERAL UNIVERSITARIO REQUENA (CV)	ESCUDERO ALARCÓN	VICENTE	Coordinator Researcher Team
	HERNANDEZ PLATERO	SONIA	Field Researcher
	PEREZ BALLESTEROS	MANUELA	Field Researcher
	NAVARRO ORTÍ	LOURDES	Field Researcher
	SORIANO FERRAGUT	CARMEN	Field Researcher
HOSP UNIVERSITARI I POLITÈCNIC LA FE VALENCIA (CV)	VIERA RODRÍGUEZ	VERÓNICA	Coordinator Researcher Team
	VIVAS BROSETA	ANA MARÍA	Field Researcher
	BAHAMONTES MULI	AMPARO	Field Researcher
	CABALLERO MARTÍ	AMPARO	Field Researcher
	CORREA VIVAS	AMPARO	Field Researcher
	SIREROL GASCÓN	CONCHA	Field Researcher
	GARCÍA TELL	FRANCISCO JAVIER	Field Researcher
	PACHECO	PILAR	Field Researcher
	MARTINEZ	Mª ÁNGELES	Field Researcher
	SEGARRA MORENO	MARTA	Field Researcher
	RODRÍGUEZ GIL	JOSE ANTONIO	Field Researcher
	ALDA CLAVERO	INMACULADA	Field Researcher
	ALARCÓN	AMOR	Field Researcher
	ÚBEDA	AMPARO	Field Researcher
	FRANCO	ASCENSIÓN	Field Researcher
	MARTÍNEZ BARCO	ENCARNACIÓN	Field Researcher
	HERNÁNDEZ ESLAVA	LOLA	Field Researcher
	AUSINA	Mª JOSÉ	Field Researcher
	CLIMENT AMORÓS	MAITE	Field Researcher
	GALLEGO VALLEJO	JOSÉ	Field Researcher
	NUÑEZ	LAURA	Field Researcher
	GALVEZ	ANA	Field Researcher
	LLARENA	ESTHER	Field Researcher
	GARCÍA	ENCARNACIÓN	Field Researcher
	MARTÍNEZ	JUANA	Field Researcher
	MONZÓN SORIANO	JOSEFA	Field Researcher
	GIRBÉS	TINA	Field Researcher
	BONET ESCRIBÁ	VICENTA	Field Researcher
	MARTI GONZALEZ	CONCEPCIÓN	Field Researcher
	CORREA VIVAS	M. AMPARO	Field Researcher
	VILLANUEVA	ALICIA	Field Researcher
	SANZ BERMEJO	ANA MARÍA	Field Researcher
	RIBES	RAQUEL	Field Researcher

	LLOBAT	VIRGINIA	Field Researcher
	GUARDIOLA SABATER	MARIO	Field Researcher
	GONZALEZ	Mº JOSÉ	Field Researcher
	MEDINA	JULIANA	Field Researcher
	CASTRO	JOSE MANUEL	Field Researcher
	VENEGAS VENEGAS	M JESÚS	Field Researcher
	VILLALON ROMERO	NOELIA	Field Researcher
	MARTINEZ ROSA	MAR	Field Researcher
	FUENTES	DOLORES	Field Researcher
	RODRÍGUEZ SEGURA	DOLORES	Field Researcher
	DURAN	NURIA	Field Researcher
	LUZÓN	ALEJANDRO	Field Researcher
HOSPITAL ARNAU DE VILANOVA (CV)	PANIAGUA MOLINER	ALICIA	Coordinator Researcher Team
	JIMENEZ GARCIA	FRANCISCA	Field Researcher
	LÓPEZ CASORRAN	MAITE	Field Researcher
	SOLAZ MARTINEZ	VICENTA	Field Researcher
	MONFORT MASIA	SONIA	Field Researcher
	VILA AGUILAR	REMEDIOS	Field Researcher
	SANCHIS FITO	REYES	Field Researcher
	MARCO MONTERO	ESPERANZA	Field Researcher
	MERINO	MIGUEL	Field Researcher
	HERNÁNDEZ ANDRES	Mª SOLEDAD	Field Researcher
	ALARCON ROIG	JUAN CARLOS	Field Researcher
	BEA ROIG	LAIA	Field Researcher
	PALES GONZALEZ	SONIA	Field Researcher
	VIZCAINO OCAÑA	ROCIO	Field Researcher
HOSPITAL COMARCAL VINARÒS (CV)	MOGARRA CAMOS	KATIA	Coordinator Researcher Team
	SANZ FORNER	ROSANA	Coordinator Researcher Team
	VILLALONGA SUBIRATS	GABRIELA	Field Researcher
	VILLALONGA ORTI	LEONOR	Field Researcher
	MONTSERRAT GUIMERA	PILAR	Field Researcher
	VALLES VALLES	LLUISA	Field Researcher
	FERNANDEZ BARRIENTOS	LAURA	Field Researcher
HOSPITAL DE LLIRIA (CV)	LOPEZ MIRALLES	CRISTINA	Coordinator Researcher Team
	PASTOR CAMPOS	SONIA	Field Researcher
	TAMARIT SALVADOR	CARLOS	Field Researcher
	GONZALEZ BIOSCA	SUSANA	Field Researcher
	MULLOR NAVARRO	Mª JOSÉ	Field Researcher
	BLAZQUEZ LOZANO	Mª JULIA	Field Researcher
	RAMOS ASENSIO	CARMEN GRACIA	Field Researcher
	BORI TORRES	REME	Field Researcher
	GARCIA MUÑOZ	CARMEN	Field Researcher
	ORERO VIDAGANY	ROSA	Field Researcher
	TARIN NAVARRO	RAQUEL	Field Researcher
	MORENO GUZMAN	ESTHER	Field Researcher
	POMER CASTILLO	RAFAEL	Field Researcher
	SANGÜESA SANZ	Mª ROSARIO	Field Researcher
	ESTEVE GASCÓ	AMPARO	Field Researcher
	REILLO REDON	BEATRIZ	Field Researcher
	PIQUERAS PASCUAL	RAQUEL	Field Researcher
	COLOMA DUCTOR	ALFONSO	Field Researcher
HOSPITAL DE MANISES (CV)	OLIVER CARAVACA	JUAN JOSÉ	Coordinator Researcher Team
	PALERO CASTELLO	CLAUDIA	Field Researcher
	MONTES GOMEZ	CARMEN	Field Researcher
	ROMERO MARTINEZ	IRENE	Field Researcher
	ANDREU MANDINGORRA	VICENTE	Field Researcher
	CORTES VALLDECABRES	MARIA DEL CARMEN	Field Researcher
	SANCHEZ GARCIA	Mª CARMEN	Field Researcher
	MARTINEZ MARCOS	NOEMI	Field Researcher
	GARCIA ORDOÑEZ	MONTSERRAT	Field Researcher
	BUENO RODRIGUEZ	ARANTXA	Field Researcher
	PEREZ ALBERO	MARIOLA	Field Researcher
	AMAYA PIRIS	BARBARA	Field Researcher
	VALERA LLORIS	RAQUEL	Field Researcher
	RAMOS PERONA	JESUS	Field Researcher
	OLMOS GARCIA	PAULA	Field Researcher
	CAMPO JIMENEZ	GONZALO	Field Researcher
	TALAVERA HERREROS	ESTHER	Field Researcher
	CRUZ NAVARRO	DAVID	Field Researcher
	AGUILAR DUBAL	CRISTOBAL	Field Researcher
	CATALÁ POVEDA	M TERESA	Field Researcher
	PLATERO ARMERO	JOSÉ LUIS	Field Researcher
	DIAZ MORRIO	AIDA	Field Researcher
HOSPITAL DE SAGUNTO (CV)	ROYO CALVO	ASUNCIÓN	Coordinator Researcher Team
	TORTOSA RUBIO	CLARA	Field Researcher
	ALONSO BENAVENT	MATILDE	Field Researcher
	CARAÑANA ABRIL	ANA	Field Researcher
HOSPITAL DENIA (CV)	PRATS CATALA	TERESA	Coordinator Researcher Team
	RUBIO PERIS	MARIA CARMEN	Field Researcher
	ESPIN ABELLAN	CATALINA	Field Researcher
	CABEDO PASTOR	OSCAR	Field Researcher
	DE LA HOZ RECIO	CARMEN	Field Researcher
	PEREZ COBO	FRANCISCO JOSÉ	Field Researcher
	GARRIDO SÁNCHEZ	JESSICA	Field Researcher
	VALDIVIA PÉREZ	ANTONIO	Field Researcher
	VILLENA DOLON	ALICIA	Field Researcher
	VINUESA MIGUEL	ROCIO	Field Researcher
HOSPITAL DR MOLINER (CV)	ANDRES CHECA	DANIEL	Coordinator Researcher Team
	PLANAS DOLS	ISABEL	Coordinator Researcher Team
	POLO MIGUEL	ASUNCION	Coordinator Researcher Team
	MARTINEZ CAPELLA	ROSA MARIA	Field Researcher
	JIMENEZ PEREZ	ALMUDENA	Field Researcher
	QUIRÓS MARÍN	CANDIDA	Field Researcher

HOSPITAL FRANCES DE BORJA GANDIA (CV)	PALAU GOMAR	ANA	Coordinator Researcher Team
	MORENO MENGUAL	CARMEN	Field Researcher
	BENAVENT PEIRO	ROSA	Field Researcher
	YORDANOVA	SNEZHANA GEORGIEVA	Field Researcher
	LOPEZ CUENCA	CRISTINA	Field Researcher
	ALBEROLA ARROYO	RUBEN	Field Researcher
	FERNANDEZ MARTINEZ	ALICIA	Field Researcher
HOSPITAL GENERAL D´ONTINYENT (CV)	CEPERO DEL CASTILLO	Mª JOSE	Coordinator Researcher Team
	ALONSO VIDAL	LUÍS ENRIQUE	Field Researcher
	SOLER GARCÍA	MERCEDES	Field Researcher
	VAELLO PASCUAL	CHARO	Field Researcher
	BODI NAVARRO	MARIA MARIOLA	Field Researcher
HOSPITAL GENERAL UNIVERSITARI D´ELX (CV)	PEREZ ALEDO	FRANCISCO JAVIER	Coordinator Researcher Team
	PASTOR MARTINEZ	MARIA	Field Researcher
	MIRAS GARCIA	MAR	Field Researcher
	HUERTAS LINERO	CRISTINA	Field Researcher
	MONTIEL FUENTES	DAVID	Field Researcher
	SANCHEZ LOPEZ	ANA CARIDAD	Field Researcher
	SOLER MARTINEZ	RAQUEL	Field Researcher
	SARMIENTO SEMPERE	RUTH	Field Researcher
	VICENTE PIÑOL	JORGE	Field Researcher
	PÉREZ SOLER	MONTSERRAT	Field Researcher
	MORANTE MARTINEZ	JOAQUIN	Field Researcher
	PAVIA MIRALLES	VANESA	Field Researcher
	SIRVENT LÓPEZ	LOLA	Field Researcher
	GARCÍA CASTELLÓ	CRISTINA	Field Researcher
HOSPITAL GENERAL UNIVERSITARIO ELDA (CV)	CREMADES BERNABEU	JOSE ANTONIO	Coordinator Researcher Team
	CREMADES BERNABEU	JA	Coordinator Researcher Team
	DELTELL GONZALEZ	YOLANDA	Coordinator Researcher Team
	HERAS CARCELEN	CARLOS	Field Researcher
	LOPEZ	PATRICIA	Field Researcher
	JUSTAMANTE BELLOD	ELIA	Field Researcher
	PLAZA GARCIA	ELENA	Field Researcher
	GOMEZ LOPEZ	VANESA	Field Researcher
	SERRANO CARRASCO	BEATRIZ	Field Researcher
	BELLOD GUILLEN	JOSE MIGUEL	Field Researcher
	MIRA PEREZ	Mª CONSUELO	Field Researcher
	SAEZ HERNANDEZ	ANA BELEN	Field Researcher
	TOMAS MARIA	MARIA VIRTUDES	Field Researcher
	GONZALEZ MIRALLES	ESTER	Field Researcher
	LORENZO MUÑOZ	RAUL	Field Researcher
	GALIANA MARTINEZ	ISIDRO JAVIER	Field Researcher
	PASCUAL FERRIZ	PABLO	Field Researcher
	PENADES CANDELA	MARINA	Field Researcher
	VIVES CASTELLANO	ALMUDENA	Field Researcher
	PUCHE RIBERA	ESTHER	Field Researcher
HOSPITAL GENERAL UNIVERSITARIO CASTELLON (CV)	LÓPEZ BENET	SUSANA	Coordinator Researcher Team
	LAZARO GIL	ROSA	Field Researcher
	ANDREU PEJO	LAURA	Field Researcher
	CAPDEVILA EJARQUE	MARTA	Field Researcher
	MONFORT GONZALEZ	Mª TERESA	Field Researcher
	REMOLAR QUINTANA	Mª JOSE	Field Researcher
	REDON BADENES	Mª ANGELES	Field Researcher
	REMOLAR GRIFO	Mª AVELLA	Field Researcher
	SEGARRA GOSALVES	OFELIA	Field Researcher
	NAVARRO GARCIA	PILAR	Field Researcher
	ROCA AGUILAR	Mª DOLORES	Field Researcher
	MARTINEZ GIMENEZ	VANESA	Field Researcher
	SORNI GAVILA	ISABEL	Field Researcher
	TORRENT RAMOS	PATRICIA	Field Researcher
	RUEDA LACRUZ	BEGOÑA	Field Researcher
CONSORCIO HOSP GENERAL UNIVERSITARIO VALENCIA (CV)	JIMENEZ LORENTE	M LUISA	Coordinator Researcher Team
	RAFAEL	ESTEVE BOSCH	Field Researcher
	CHOVER SIERRA	ELENA	Field Researcher
	CAMPOS RUBIO	FRANCISCO	Field Researcher
	FOLGADO ROIG	JOSE	Field Researcher
	IRUELA	JORGE	Field Researcher
	APARICIO SANCHEZ	ROSARIO	Field Researcher
	GONZALEZ GARCIA	PILAR	Field Researcher
	GARRIDO CASTELLO	MARGARITA	Field Researcher
	HERRAIZ LOPEZ	PILAR	Field Researcher
	FAYOS JOVER	JESUS	Field Researcher
	GALINDO GARCIA	MARTA	Field Researcher
	SANTIAGO TORTAJADA	ISABEL	Field Researcher
	GINER BONORA	INMACULADA	Field Researcher
HOSPITAL LA MAGDALENA (CV)	PEREZ RODRIGUEZ	Mª PILAR	Coordinator Researcher Team
	NOTARI MEZQUITA	Mª MERCEDES	Field Researcher
	RUIZ GARATE	Mª TERESA	Field Researcher
HOSPITAL LA PEDRERA (CV)	LLORCA LLORCA	RAÚL	Coordinator Researcher Team
	SIMÓ NOGUERA	INMA	Field Researcher
	PLAZA LANGREO	CLARA	Field Researcher
HOSPITAL LLUÍS ALCANYÍS (CV)	GARCÍA ESTEVE	JOCABED	Coordinator Researcher Team
	FERNANDEZ MARTINEZ	ALICIA	Coordinator Researcher Team
	BELTRAN HERRERO	MARIA LUISA	Field Researcher
	AVILA MARTINEZ	DAMIAN	Field Researcher
	MEJIAS TOMAS	MONTSERRAT	Field Researcher
	ARGENTE CALATAYUD	JOSÉ VICENTE	Field Researcher
	GARCÍA ALAPONT	RAFAEL	Field Researcher
HOSPITAL MARINA BAIXA (CV)	CASTILLO GOMEZ	CARMEN	Coordinator Researcher Team
	HERNANDEZ VIDAL	PEDRO ANGEL	Field Researcher
	SANCHEZ GARCIA	ROSA MARIA	Field Researcher

	GALARRETA VIDAURRE	MARIA CRUZ	Field Researcher
	ZORRILLA PINEL	ANA MARIA	Field Researcher
	ZAMORA HEREDIA	FRANCISCA	Field Researcher
	SIRVENT SANTAMARIA	MONSERRAT	Field Researcher
HOSPITAL PADRE JOFRE (CV)	GIL VILA	M. YOLANDA	Coordinator Researcher Team
	MONZÓN MIRASOL	M. LUZ JUANA	Field Researcher
HOSPITAL SANT VICENT DEL RASPEIG (CV)	MORALES ESPINOSA	CONCEPCIÓN	Coordinator Researcher Team
	CUARTERO ABRIL	Mª ANGELES	Field Researcher
	ALCOCER GOMIS	MARIA BELEN	Field Researcher
	LÓPEZ GONZÁLEZ	ESTHER	Field Researcher
	PARAISO GONZALEZ	AMAYA	Field Researcher
	CUEVAS HOPMAN	AROA MARÍA	Field Researcher
	ALMARCHA UBEDA	INMACULADA	Field Researcher
	PASTOR TOMÁS	RAÚL	Field Researcher
	DOMENECH MARIA	LOURDES	Field Researcher
	MONTESINOS BUTRON	PILAR	Field Researcher
	BUADES MOLINA	RAQUEL	Field Researcher
	MORENO MORALES	FRANCISCO JESUS	Field Researcher
HOSPITAL UNIVERSITARI DE LA PLANA (CV)	MARTÍN PAREJA	MANUEL	Coordinator Researcher Team
	VIDAL MARTINAVARRO	GISELA	Field Researcher
	RUS MATA	SARA	Field Researcher
	SORIA FERNANDEZ	GISELA	Field Researcher
	GARCES MARTÍ	Mª JOSÉ	Field Researcher
	RUS MATA	SARA	Field Researcher
	MARTÍN PAREJA	MANUEL	Field Researcher
	PILAR	PESUDO FALCO	Field Researcher
HOSPITAL UNIVERSITARI SANT JOAN. ALACANT (CV)	AGUILERA MARTINEZ	ROSA	Coordinator Researcher Team
	FERNANDEZ GONZAGA	CRISTINA	Field Researcher
	VERDÚ PASTOR	CONCEPCIÓN	Field Researcher
	LÓPEZ BARBERA	TERESA	Field Researcher
	BLASCO MIGUEL	JOSE ALBERTO	Field Researcher
	GUEVARA VERA	EMILIA	Field Researcher
	MOLINA PICÓ	NOELIA	Field Researcher
	MONTEJANO BALLESTER	FERNANDO	Field Researcher
	CASADO LLAVONA	CARMEN	Field Researcher
	BUSTOS MARTINEZ	ROSA	Field Researcher
	SANABRIA GARCÍA	RAQUEL	Field Researcher
	QUER RAMÓN	SANTIAGO	Field Researcher
	ESPARCIA NOVOA	Mª JOSÉ	Field Researcher
HOSPITAL UNIVERSITARIO DE LA RIBERA (CV)	GOMIS BALDOVI	SONIA	Coordinator Researcher Team
	LLANES DOMINGO	JOSE VICENTE	Field Researcher
	ZAFRA SOLAZ	CARMEN	Field Researcher
	MORENO CAZALLA	SILVIA	Field Researcher
	MONEDERO ALONSO	LUIS	Field Researcher
	PRIMO CHAQUES	JOSE ANDRÉS	Field Researcher
	ORQUIN NAVARRO	MARISOL	Field Researcher
	MARTINEZ LLOPIS	BEATRIZ	Field Researcher
	AMOROS CANTERO	AURORA	Field Researcher
	PEÑARROCHA AROCAS	SARA	Field Researcher
	CORACHÁN DOMINGUEZ	FLORENTINA	Field Researcher
	LANGA LLOPIS	MONICA	Field Researcher
	OYA BELENGUER	MIRIAM	Field Researcher
	RODRIGUEZ HERRERA	ANGELES	Field Researcher
	MARTI MERINO	Mª TERESA	Field Researcher
	GARCÍA-NOBLEJAS JULIÁ	PABLO	Field Researcher
	BELTRÁN BRINES	RAQUEL	Field Researcher
HOSPITAL UNIVERSITARIO DEL VINALOPO (CV)	FERNANDEZ DE MAYA	JOSÉ	Coordinator Researcher Team
	GIL CARBONELL	MARIA JOSE	Coordinator Researcher Team
	GARCIA PERLA	PABLO	Field Researcher
	MONASOR	DAVID	Field Researcher
	LEAL LOPEZ	MARIA DEL CARMEN	Field Researcher
HOSPITAL UNIVERSITARIO DR PESET VALENCIA (CV)	HURTADO NAVARRO	CLARA	Coordinator Researcher Team
	MARTINEZ COLETO	ELENA	Field Researcher
	PAULO BARATO	JUAN ANTONIO	Field Researcher
	ABELLAN BALLESTEROS	DOLORES	Field Researcher
	LLÀCER VINYES	RICARDO	Field Researcher
	ORTIZ DE ELGUEA	ENRIQUE	Field Researcher
	GÁLVEZ PRAT	JOSE FRANCISCO	Field Researcher
	MARTINEZ MORA	MARIA TERESA	Field Researcher
	MARTÍNEZ JUAREZ	MARÍA ASUNCIÓN	Field Researcher
	ORQUÍN LLOPIS	MARÍA JOSEFA	Field Researcher
	SAN JOSÉ PLANELLS	RAMÓN	Field Researcher
	TORRIJOS DE LA TORRE	MARÍA AMPARO	Field Researcher
	VALERA TALAVERA	DUNIA	Field Researcher
	MERCADO MARTÍNEZ	AMPARO	Field Researcher
	SANZ GIMENO	SARAY	Field Researcher
	ROMERO MORÁN	ANGELA	Field Researcher
	BALLESTER ARIAS	ANTONIO RAMÓN	Field Researcher
	GRACIA MONESMA	JOSE ALFREDO	Field Researcher
	SOLER CARBÓ	RAFAEL ANDRÉS	Field Researcher
	NUÑEZ LÓPEZ	ELIECER	Field Researcher
	AGUADÉ GARCÍA	CARLOS	Field Researcher
	MARTINEZ GARCIA	ISRAEL	Field Researcher
	VINAT COLLADO	ROSA	Field Researcher
	VIZCAINO SANCHIS	ESTRELLA	Field Researcher
	TOMAS GARCÍA	VICENTE	Field Researcher
	LUCAS FERNANDEZ	ANA	Field Researcher
HOSPITAL UNIVERSITARIO TORREVIEJA ALICANTE (CV)	LEAL LOPEZ	MARIA CARMEN	Coordinator Researcher Team
	GIL CARBONELL	MARÍA JOSÉ	Field Researcher
	CABRERA RAMIREZ	ALBERTO	Field Researcher
	BERENGUER ORTUÑO	SENÉN	Coordinator Researcher Team

HOSPITAL VEGA BAJA ORIHUELA (CV)	GAMAYO SERNA	ANA	Field Researcher
	MENGUAL GOMEZ	PILAR ADELA	Field Researcher
	BOTELLA MATEO	MARIA DEL CARMEN	Field Researcher
	TAFALLA TORRES	JOSE MANUEL	Field Researcher
	RUIZ VIDAL	ANA ROSA	Field Researcher
	BERNABE CASES	OLAYA	Field Researcher
	SAURA RIVES	ESPERANZA	Field Researcher
HOSPITAL VIRGEN DE LOS LIRIOS ALCOY (CV)	MARTINEZ MUNTO	RAQUEL	Coordinator Researcher Team
	DOMENECH CLIMENT	NURIA	Field Researcher
	VALDELVIRA GIMENO	BEATRIZ	Field Researcher
	RUBIO GARCÍA	LLUIS	Field Researcher
	MONTAVA TOMAS	JUAN VICENTE	Field Researcher
	BRAVO MIRÓ	JORDI	Field Researcher
COMPLEJO HOSPITALARIO UNIVERSITARIO INSULAR MATERNO INFANTIL (CANARIAS)	NAYA ESTEBAN	JOAQUÍN JOSE	Coordinator Researcher Team
	DÍAZ GONZALEZ	CANDELARIA DE LA MERCED	Field Researcher
	CAMPOS GUERRA	MABEL	Field Researcher
	CLAVEL LAGO	ANTONIO	Field Researcher
	DOMINGUEZ CASTELLANO	ELIZABETH	Field Researcher
	BERMUDEZ JIMÉNEZ	MAITE	Field Researcher
	DOMÍNGUEZ MONZÓN	MILA	Field Researcher
	GONZALEZ MEDINA	RAÚL	Field Researcher
	GUILLÉN MEDEROS	NOEMÍ	Field Researcher
	MEDINA ARRIBAS	YURENA	Field Researcher
	MONTOYA GARRIDO	Mª JOSEFA	Field Researcher
	MORAL ARROYO	FERNANDO	Field Researcher
	PALMÉS QUEVEDO	RICARDO	Field Researcher
	PANADERO GARCÍA	TAMARA	Field Researcher
	RAMÍREZ FALCÓN	PATRICIA	Field Researcher
	RAMÍREZ SANTANA	ESTEFANÍA	Field Researcher
	SANCHEZ ARBELO	ALEXIS	Field Researcher
	SANTANA LEÓN	ANA	Field Researcher
	SANTANA PÉREZ	NOELIA	Field Researcher
	SORIA MARTÍN	DARA	Field Researcher
	YANEZ SANTANA	YURENA	Field Researcher
	ZERPA SANTANA	RAFAEL	Field Researcher
	GONZÁLEZ GONZÁLEZ	CAROLINA	Field Researcher
	SANTANA GONZALEZ	MARTA	Field Researcher
	ÁLVAREZ VALERÓN	BEATRIZ	Field Researcher
	BAEZ BAEZ	PATRICIA	Field Researcher
	BATISTA ARTEGA	ÁNGELES	Field Researcher
	PAREDES LÓPEZ	LUIS	Field Researcher
	MAYOR MONZÓN	YAIZA	Field Researcher
	GORDILLO CORONADO	LAURA	Field Researcher
	VEGA MEDEROS	METEIMBA	Field Researcher
	GONZALEZ CABRERA	MARTA	Field Researcher
	ÁLVAREZ RAMOS	CANDY	Field Researcher
	ALONSO MARRERO	CARMEN	Field Researcher
	HERRERA HERNÁNDEZ	PEDRO	Field Researcher
	MACHÍN LOYZANCE	GAEL	Field Researcher
	FERNÁNDEZ TURRADO	ELISA	Field Researcher
	SANTANA NARANJO	CARIDAD	Field Researcher
	GONZÁLEZ TARBAY	RAFAEL	Field Researcher
	DEL ROSARIO CASTELLANO	MARTA	Field Researcher
